# Recommendations for Physical Exercise as a Strategy to Reduce Problematic Use of the Internet and Digital Devices: A Perspective

**DOI:** 10.3390/ijerph22050753

**Published:** 2025-05-10

**Authors:** Christel García-Ortiz, Miriam Lorenzo-González, Javier Fernández-Sánchez, Víctor Solano-Lizcano, Juan Del Coso, Daniel Collado-Mateo

**Affiliations:** Sport Sciences Research Centre, Rey Juan Carlos University, 28943 Fuenlabrada, Spain; c.garciao.2018@alumnos.urjc.es (C.G.-O.); miriam.lorenzo@urjc.es (M.L.-G.); javier.fernandezsa@urjc.es (J.F.-S.); v.solano.2020@alumnos.urjc.es (V.S.-L.)

**Keywords:** sedentary behavior, behavioral addiction, physical activity, mental health promotion, nature exposure

## Abstract

Excessive use of the Internet and digital devices has become a growing public health concern, contributing to mental health issues, sedentary lifestyles, and decreased well-being. Despite the increasing prevalence of digital overuse, there is no consensus on effective interventions to mitigate its impact. This article aimed to provide an updated perspective on current research into problematic use of digital devices and the Internet, and to explore the potential of physical exercise as a key strategy in prevention and treatment programs aimed at reducing such use. Based on the two previous objectives, this study also aimed to provide author-informed recommendations for digital detox interventions, supporting the inclusion of regular exercise, particularly outdoor exercise in natural environments, as a central component of such programs. Thirteen recommendations for physical exercise programs to be included as core parts of digital detox are suggested: (a) the type of physical exercise selected according to the participant’s motivations, (b) exercise intensity and volume adapted to the participant’s characteristics, (c) the avoidance of digital devices during exercise, (d) a duration of ≥12 weeks and ≥3 days/week, (e) the integration of other physical activities for a more active lifestyle, (f) the enhancement of mental health as a main goal, (g) collaborative and competitive physical exercise and sports, (h) the participant’s awareness of the improvements through feedback and information, (i) mindful activities, (j) outdoor physical activities and exercise, especially in natural environments, (k) conducted by multidisciplinary teams, (l) assessments using validated tests and scales, and (m) evaluation of long-term effects. In summary, this perspective article supports the inclusion of physical exercise as a key strategy in digital detox programs by offering recommendations for intervention designs aimed at reducing problematic digital use and enhancing overall well-being in individuals who have developed maladaptive patterns of digital device use.

## 1. Introduction

The Internet and smartphones have revolutionized communication, education, and access to information, offering unprecedented opportunities for connectivity, learning, and entertainment. In the context of public health, access to the Internet and smartphones has provided significant benefits by enhancing access to reliable medical information, supporting health resources, promoting health education, and empowering individuals to make informed health decisions. Along with their numerous benefits, the use of smartphones, the Internet, and social media has increased significantly in recent years, with daily online activity, texting, and social media engagement surpassing time spent on print media, books, TV, and movies [1]. The use of the Internet and social media has gone beyond entertainment to become fundamental tools for daily living and work [2]. While these tools offer numerous benefits, their excessive use can become overly compelling, sometimes leading to maladaptive behaviors. This growing concern poses a significant health challenge that could potentially reach pandemic levels, especially for young adolescents, as they are particularly vulnerable. The rising trend of excessive internet use and connected device engagement aligns with a progressive increase in physical inactivity and prolonged indoor time, a pattern that was further accelerated by the COVID-19 pandemic [3].

In this context, excessive smartphone use and prolonged digital exposure have been linked to an increase in mental health issues [4]. In recent years, various concepts have emerged to describe the problematic use of smartphones, the Internet, and social media. One such concept is maladaptive escapism, which refers to the use of digital devices and online platforms as a means to avoid or escape reality. This phenomenon is particularly prevalent among adolescents and young adults, often interfering with daily life and contributing to excessive use or even addiction [5]. To deal with this maladaptive behavior, digital detox strategies have been proposed in the scientific literature [6]. Digital detox is a broad concept that encompasses different actions to promote well-being by voluntarily restricting the use of smartphones, the Internet, or social media [6]. Despite the potential benefits of digital detox strategies, there is a lack of specific guidelines on how they should be implemented, and there is limited evidence on whether incorporating physical exercise could enhance their effectiveness.

The scientific community has already recognized problematic internet and digital device use as a public health concern, prompting various initiatives such as the aforementioned digital detox programs, aimed at mitigating its impact. However, a major gap in the scientific literature is the lack of consensus on terminology and the inaccurate use of language and diagnostic terms [7]. Although terms like ‘digital addiction’, ‘internet addiction’, ‘problematic social media use’, ‘smartphone addiction,’ ‘problematic online gambling,’ or ‘excessive smartphone use’ are frequently used interchangeably in the scientific literature, they still lack officially recognized diagnostic criteria [8]. In this regard, ‘internet gaming disorder’ is the only problematic digital behavior included in the Diagnostic and Statistical Manual of Mental Disorders (DSM-5) [9]. The absence of a standardized and universally accepted framework for defining digital addictions significantly hampers the development of consistent prevention strategies and evidence-based treatment interventions.

In this context, the scientific literature has frequently extrapolated the characteristics and consequences of drug addictions to digital addictions, given observed similarities in the brain connectivity and dopamine responses [10]. However, this leads to a wrong conceptualization since there are several relevant differences that were discussed by Panova et al. [7]. A key distinction lies in the differentiation between ‘excessive use’ and ‘problematic use’. In modern society, digital devices are integrated into nearly every aspect of daily life, and many common activities such as listening to music, messaging friends or family, or using navigation apps while driving are not inherently problematic. Therefore, when discussing ‘tolerance’ or ‘withdrawal’ in digital addictions, it is essential to consider not only the amount of time spent using digital devices, but also the underlying motivations for use and the associated psychological or functional consequences [8,11]. This absence of standardized terminology and diagnostic criteria for digital addictions, combined with a limited number of studies focused on mitigating their impact, hinders the establishment of a consensus on effective prevention and treatment strategies for these problematic behaviors [8]. Panova et al. [7] recommend replacing the term ‘addiction’ with ‘problematic use’ or ‘problematic behavior’ when describing maladaptive behaviors associated with digital device use, as this terminology more accurately reflects what existing scales and questionnaires measure. Following this recommendation, we will use this terminology in the current study and recommend this usage in future studies.

**Figure 1:**
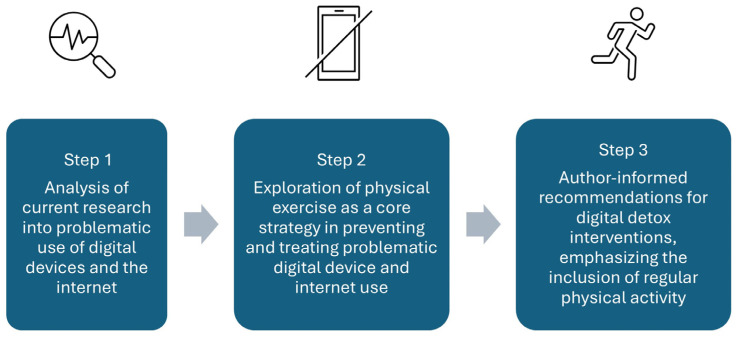
Flowchart with the three-step aim of this perspective article on recommendations for physical exercise as a strategy to reduce problematic use of the Internet and digital devices.

Although the characteristics and consequences of problematic digital device use may not be directly comparable to those observed in substance addictions, its high prevalence—surpassing that of many common social drugs—and significant impact on daily life and mental health underscore the importance of addressing this issue in the coming years [12]. Thus, this article aimed to provide an updated perspective on current research into problematic use of digital devices and the Internet, and to explore the potential of physical exercise as a key strategy in prevention and treatment programs aimed at reducing such use. Based on the two previous objectives, this study also aimed to provide author-informed recommendations for digital detox interventions, supporting the inclusion of regular exercise, particularly outdoor exercise in natural environments, as a main component of such programs (Figure 1).

## 2. Problematic Use of the Internet and Digital Devices

The problematic use of digital devices is increasingly recognized as a growing concern, particularly among adolescents and young adults [13,14]. According to global estimates by Meng et al. [15], over 25% of individuals exhibit smartphone addiction, 17.42% struggle with social media addiction, and 14.22% are addicted to the Internet. Furthermore, social networks and electronic devices tend to exacerbate social stressors, such as FoMo (Fear of Missing Out) or nomophobia (fear of being without access to a smartphone) [16,17]. Previous studies have shown prevalences close to 100% for nomophobia in university students, along with potential negative consequences for behavior, physical and mental health, as well as academic performance [18,19,20,21]. This large prevalence and the potential negative consequence of nomophobia is especially worrying among children and adolescents aged 12 or more, which has led to the notion that it is becoming one of the most outstanding health issues in the 21st century [22].

Apart from age differences, some gender variances have been identified, with men being more prone to video game and gambling addictions compared to women [23], while women may be more inclined to have higher levels of addiction to social networks and smartphones [24,25]. Previous studies have shown that female adolescents had a higher prevalence of passive interaction with social media (browsing or consuming content), which may be positively related to eating disorders [26]. On the other hand, a higher frequency of active social media interaction (posting content) may be associated with higher mental health issues regardless of gender [27].

These discrepancies in terms of prevalence and consequences highlight the relevance of the gap in the lack of consensus on terminology and the inaccurate use of language and diagnostic terms. For instance, nomophobia must be understood under the umbrella of the problematic digital device use but should not be included as “digital addiction”, since the prevalence of nomophobia is around four times higher than the prevalence of smartphone addiction. Similarly, including active and passive interaction as the same variable (i.e., under the umbrella of social network addiction) may lead to bias in the interpretation of consequences and changes in research.

Considering the large prevalence and the multidimensional health consequences of digital addiction, an interdisciplinary approach that integrates public health initiatives, educational programs, and digital literacy campaigns is required. Various interventions have been explored and documented in the scientific literature, employing different strategies to mitigate problematic digital use. Among them, digital detox programs—primarily focused on limiting or restricting smartphone, social media, and internet use—remain one of the most common behavior-modification approaches. However, inconsistent findings and a significant lack of consensus regarding definitions and measurement criteria hinder the ability to draw solid conclusions [28], underscoring the need for further research. In this regard, not every digital detox program will lead to meaningful positive effects. A recent systematic review showed mixed results, with some studies reporting positive effects and others revealing neglective or negative effects [29]. This systematic review also showed that this heterogeneity is even more worrying if we are looking for long-term effects, which may involve the acquisition of healthier digital and lifestyle habits. One of these lifestyle habits that has gained attention in the scientific literature is physical activity. Previous research supports that engaging in physical activity and structured exercise programs or sports reduces time spent on sedentary behaviors, such as smartphone use or social media scrolling, while excessive phone use can, in turn, limit the time dedicated to exercise [30]. Thus, from the point of view of the authors, interventions aimed to reduce the problematic use of the Internet and digital devices should consider the potential long-term benefits of physical exercise.

## 3. The Role of Physical Exercise in Promoting Well-Being and Reducing Problematic Digital Device Use

Physical activity and exercise are associated with higher levels of subjective well-being [31], and they are widely known to improve physical function, cardiometabolic health, bone density, and adiposity profile—factors that reduce mortality and enhance health-related quality of life [32,33]. Additionally, regular exercise fosters social connections and a sense of community, particularly in group-based settings, which contributes to emotional support and a shared sense of achievement, further enhancing mental health outcomes [34]. Among the physiological benefits of physical activity, the reduction of cortisol levels and the release of dopamine and serotonin play a crucial role in enhancing mental health and overall well-being. These neurochemical changes are directly associated with stress reduction, mood regulation, and the experience of pleasure [35,36,37].

Although the benefits of exercise for well-being are evident regardless of the type or intensity of exercise [38], a regular, sustained participation in physical exercise is essential to achieve significant mental health enhancements and this is a major challenge since adherence to physical exercise is often poor and strategies aimed to facilitate the integration of exercise as part of the lifestyle are needed [39]. In this regard, several key factors associated with adherence to physical exercise have been previously reported in different populations, highlighting the need to consider aspects such as individualization, supervision, feedback, and goal setting during the design and implementation of exercise programs [40]. While these factors are critical for improving adherence and maximizing the health benefits of exercise, they may also play a role in understanding the complex relationship between physical activity and various forms of problematic digital behavior, including excessive use of smartphones, the Internet, and gaming or gambling-related activities.

Regarding the benefits of exercise for problematic use of the Internet and digital devices, previous research has consistently found an inverse association between problematic smartphone use and individual levels of physical activity [41,42,43]. Specifically, individuals with higher levels of smartphone addiction or problematic use tend to be less physically active. Similar inverse relationships have been observed in studies examining problematic internet use [44] and excessive social media engagement [45,46,47]. Furthermore, in the framework of digital addictions and problematic digital behaviors, such as gaming, including offline and online videogames, and online gambling, are other activities that could have negative consequences for the users. The link between being active and being addicted to playing games is still poorly explored, but a positive relation has been previously observed [48], indicating that higher levels of physical activity may correlate with problematic gaming. As for gambling disorders, they have also been directly related to physical activity, showing that those with higher levels of physical activity are more prone to engage in gambling [49]. One possible explanation could be that people who are generally more active are also more likely to enjoy watching sports, gambling on sporting events, and playing sports-based video games [49]. However, Angelo et al. [50] found a significant reduction in gambling behaviors after an exercise program. In this sense, further research is needed to gain a better understanding of these types of digital addictions. Despite the varying associations between physical activity levels and different forms of digital addiction and online gambling, the substantial body of evidence supporting the mental health benefits of exercise and its effectiveness in addressing other types of addiction leads the authors of this study to recommend increasing physical activity through regular exercise as a promising strategy to reduce maladaptive digital device use.

Regarding exercise intensity, studies suggest that individuals engaging in intense physical exercise activities are less prone to developing problematic digital behavior compared to those participating in exercise of lower intensity [51,52]. As a result, interventions designed to promote higher physical activity levels through exercise have shown potential in mitigating digital addictive behaviors, with research suggesting that these programs should last at least 12 weeks with three to five sessions per week to be effective [53]. Liu et al. [53] systematically analyzed nine experimental studies about exercise and smartphone addiction and demonstrated that various types of physical activities—including Tai Chi, basketball, dancing, and running—can help to treat this problematic behavior. That effect may be attributed to the ability of exercise to divert attention from the digital world, encourage adaptation to real-life environments, and foster interpersonal skills such as self-control [51]. In the case of sports, they may further contribute to personal development by enhancing teamwork and communication skills, thus fulfilling individuals’ basic psychological needs [51]. These findings underscore the multifaceted benefits of structured and sufficiently intense exercise interventions, not only as a means of reducing problematic digital behaviors but also as a valuable tool for promoting healthier psychosocial functioning and overall well-being.

According to Precht et al. [54], increasing any type of daily physical activity by 30 min caused a reduction in the problematic use of smartphones. In that study, any type of exercise was acceptable, including walking, running, cycling, swimming, or weight training. However, the type of exercise performed also appears to affect its effectiveness in reducing digital addictions. Xiao et al. [55] showed that practicing Badu Anjin (a traditional Chinese Qigong exercise that consists of eight distinct movements designed to promote physical health, mental well-being, and energy flow) and basketball led to a decrease in the rates of problematic smartphone use, anxiety and loneliness among the participants who practiced these sports. Interestingly, those who practiced basketball had larger improvements, while the effects of Badu Anjin lasted longer. The authors suggested that playing basketball would lead to higher levels of dopamine due to competition and social interaction, while mindful movements of Badu Anjin could cause discipline and endurance. Similarly, the meta-analysis by Liu et al. [53] found that closed motor skill activities were associated with greater reductions in smartphone addiction compared to open motor skill activities. In contrast, other authors have suggested that open-skill sports could be the best option to mitigate addiction as they introduce several key-elements like competition, change, novelty, and social interaction, which may be associated with higher levels of dopamine and thus reducing the incidence of addictions by promoting a more balanced dopaminergic response [56,57].

Interestingly, outdoor physical exercise may be more effective than indoor activities in enhancing both physical and psychological health [58]. Activities such as running, hiking, and team sports have been linked to greater psychological benefits compared to indoor exercises, partly due to their immersive engagement with nature [59]. A recent systematic review proved that structured nature-based interventions, conducted in green or blue natural environments, effectively reduce depression and anxiety symptoms while enhancing positive affect [60]. Therefore, outdoor physical activities may be especially useful to be included in digital detox programs, combining them with balanced screen use to enhance overall well-being [61,62]. As a result, additional psychological and physical benefits can be obtained from practicing exercise in outdoor environments [63], like enhancing the capacity of individuals to reduce depression [64], as well as preventing and treating Internet addiction in adolescents [65]. However, the long-term effects of outdoor interventions still need to be investigated [66]. While more research is needed to fully understand the underlying mechanisms, the interpretation of the authors of the current study is that incorporating outdoor exercise into daily routines or intervention programs may be a simple yet effective strategy to improve both physical and psychological well-being. Encouraging activities in natural environments could consequently be particularly beneficial for mental health, offering an accessible and holistic approach to stress reduction and emotional balance, which may be useful to deal with the problematic use of the Internet and digital devices.

In summary, people with lower levels of physical activity often tend to have more maladaptive digital behaviors, as well as worse sleep quality and higher levels of stress than their peers [67]. Given these findings and from the point of view of the authors of the current study, increasing physical activity, especially through exercise conducted in the natural environment, presents itself as a potentially effective strategy for reducing problematic use of digital devices, particularly in younger populations. The existing evidence highlights the potential of maintaining high physical activity levels as both a preventive and therapeutic strategy against problematic digital behaviors, emphasizing the importance of incorporating structured exercise programs into digital detox initiatives. Based on the authors’ experience and interpretation of the current evidence, it is recommended that future digital detox interventions prioritize the inclusion of regular, structured physical activity, preferably in outdoor settings, as a practical, accessible, and multidimensional approach to support behavioral regulation, reduce maladaptive digital use, and promote long-term psychological well-being.

## 4. Recommendations for Digital Detox Programs: The Inclusion of Physical Exercise

Based on previous research, it is evident that problematic and maladaptive use of digital devices, the Internet, and social media represents a significant public health challenge. However, the current scientific knowledge on effective strategies to reduce both the prevalence and impact of this global issue remains limited and inconsistent. Although previous research has highlighted the potential benefits of psychological (including mindfulness therapy and cognitive behavioral therapy) and exercise interventions to reduce the problematic and excessive use of digital devices [56], the results are still controversial.

On this point, different digital detox programs have been conducted to reduce the problematic behavior associated with the Internet and digital devices by voluntarily restricting the use of smartphones, the Internet, or social media. However, not every type of digital detox program will lead to achieving the same effect. For instance, Walsh et al. [68] found that restricting smartphone use may lead to several benefits, such as higher life satisfaction or self-esteem, while restricting social media could have fewer benefits and some undesired consequences, such as negative emotions. Similarly, a previous meta-analysis [69] based on digital detoxification showed significant beneficial effects of this practice on well-being, while another meta-analysis by Ramadhan et al. [70] found no relevant results in well-being or in satisfaction with life in social media detoxification interventions. Thus, further research is clearly needed to better understand the adequate approach according to the characteristics and needs of the participants.

To explain this controversy, digital detox has been related to the concept of dopaminergic detox or dopamine fasting, which is based on the notion that excessive dopamine stimulation resulting from activities like social media (likes, notifications, etc.) may be associated with addictive behaviors, impulsivity, or attention deficits [71]. However, poorly designed dopamine fasting should be performed with caution due to potential negative consequences on mental and physical well-being [72]. Therefore, future studies should consider that a wrongly determined restriction in the use of the Internet or digital devices may lead to undesired consequences.

Programs aimed to detoxify from the Internet and digital devices largely differ in duration, ranging from a few days [73] to several weeks [74] and in the activities and therapeutic approaches included. Regarding protocols, some interventions restrict the use of digital devices completely or for specific time intervals (for instance, 30 min each day [73]), while others include changes in the device settings, such as turning off notifications and limiting the use of social networks [16]. However, just restricting or preventing the use of digital devices and apps may not maximize benefits. Previous studies have suggested that digital detox interventions can be more effective when combined with other therapies or activities, such as mindfulness [16], physical exercise, summer camps [75], or digital hygiene practices, encouraging interpersonal relationships and promoting technology-free activities such as retreats or outdoor physical activity and sports [74]. Thus, digital detox must be understood from a multidisciplinary perspective.

Based on the previous research and from the point of view of the authors of this article, exercise should be included in digital detox programs due to the well-known benefits in physical and mental health, the promotion of social interactions, the reduction of loneliness, stress, inadequacy, fatigue and withdrawal feelings [57,76], as well as the significant enhancement of self-esteem [77,78], which may be inversely associated with the risk of developing an addiction [79]. Furthermore, outdoor physical exercise may be recommended due to the additional benefits of spending time in natural environments and the need to disconnect from technology to connect with nature. In this regard, the relationship between the virtual and real world has changed a lot in the last few years. The traditional concept of escapism, which was based on using the virtual world to get the feelings and sensations that were not achieved in the real world, has grown so much that nowadays there is a need of an inverse escapism, where people escape from the virtual world through the engagement in activities in the real world and through the connection with nature. Although the potential benefits of physical activity and exercise in real natural environments, including green (forests, parks, gardens, etc.) or blue (lakes, rivers, seas, etc.) exercise, are significant [63], it is a major challenge, as around 55% of global population lives in urban areas [66] with scarce green spaces [66], less opportunities to practice outdoor activities and consequently higher risk of developing a problematic use of the Internet and digital devices [80]. This could be counteracted by changing the design of the cities in a way that promotes outdoor physical activity and sports practice [64] and utilizing specialized sports and health-education camps that exist in several countries [81] to focus on outdoor physical exercise and sport.

In order to extract some practical applications of the existing scientific literature on digital detox and physical exercise, the authors of the current manuscript have interpreted the previous research and provided key aspects to be considered when implementing digital detox programs, with special emphasis on physical exercise as a core component. These recommendations are presented in Table 1.

## 5. Future Perspectives and Practical Applications

The future of investigations into physical exercise and problematic digital behavior is exciting. Controversial and heterogeneous results represent an opportunity for researchers to expand knowledge and contribute to the field. In the context of digital detox, promoting healthy habits seems to be essential to achieve long-term effects, with physical activity and exercise playing a crucial role. Most existing research relies on cross-sectional studies that use questionnaires as the primary tool to examine this relationship. To establish causal links and draw reliable conclusions, future studies should incorporate longitudinal and experimental designs, assessing long-term effects and including follow-up evaluation [29]. In addition, both self-reported data and objective measures, such as smartphone usage tracking [68,82]. Additionally, further research is needed to determine the specific characteristics of physical exercise that yield optimal results by examining and comparing different frequencies, durations, intensities, and types of exercise (e.g., aerobic *versus* strength training, open-skill sports, and mindful physical activities). Moreover, the underlying mechanisms through which physical exercise influences the prevention and reduction of problematic internet and digital device use must be thoroughly explored. In the current manuscript, we provide some practical recommendations that can be considered by future research when designing and conducting digital detox programs. However, it must be noted that those recommendations are based on the comprehensive interpretation that the authors have made of the scientific literature.

Another promising topic in this field is the potential added value of outdoor exercise and physical activity in natural environments. In this way, although outdoor exercise may lead to extra benefits on the mental level and on certain symptoms such as depression, the implementation of this type of exercise program is challenging in the context of our indoor generation. The distribution of the population in urban areas and the reduced access to green or blue natural spaces in the cities are a major handicap for people to engage in these types of physical activity. Finally, policymakers and educators should consider incorporating digital literacy and wellness programs into school curricula, emphasizing balanced technology use and active lifestyles from an early age. In order to facilitate these implementations, interdisciplinary collaborations from different disciplines are essential to design innovative strategies that address the complex nature of problematic use of the Internet and digital devices in modern society.

Finally, it is important to note that most previous reviews aimed to evaluate interventions to reduce the problematic use of the Internet and digital devices have concluded that there is a relevant lack of consensus on terminology, highlighting the common confusion among terms such as ‘addiction’, ‘problematic use’, and ‘excessive use’. In this regard, based on previous research, we propose the use of ‘problematic use’ as a broader and more inclusive term that encompasses both addiction and excessive use. Since only the ‘Internet Gaming Disorder’ is currently recognized in the DSM-5, further research and standardization are needed to better define the causes, effects, symptoms, and interventions for other problematic digital behaviors to enhance prevention and treatment strategies.

## 6. Conclusions

The problematic use of the Internet and digital devices represents implications for mental health, societal well-being, and overall quality of life. In this regard, digital detox programs involving the restriction in the use of smartphones, the Internet, or social media have shown controversial but promising benefits in dealing with this global public health challenge in the modern era. However, long-term effects and optimal characteristics of this type of program need further well-designed studies. Evidence suggests that digital detox effectiveness may be enhanced by including psychological therapies (i.e., cognitive-behavioral or mindfulness therapies) and the adoption of healthy habits. Thus, increasing physical activity and exercise participation may play a key role in dealing with the problematic use of the Internet and digital devices and should be considered in digital detox interventions due to the well-known benefits of physical activity on mental health and self-esteem. Regarding the most suitable type of physical exercise, green or blue exercising and outdoor physical activities offer a promising approach to mitigate the impact of problematic use of the Internet and digital devices and promote a healthier and more balanced lifestyle. Nevertheless, the development of outdoor physical exercise programs is often limited by the urban available spaces.

Based on the authors’ interpretation of previous research, a total of 13 recommendations for physical activity programs to be included as core parts of digital detox were suggested: (a) the type of physical exercise selected according to the participant’s motivations, (b) exercise intensity and volume adapted to the participant’s characteristics, (c) the avoidance of digital devices during exercise, (d) exercise interventions with a minimum length of 12 weeks and at least three sessions per week, (e) the integration of physical activity for a more active lifestyle, (f) the enhancement of mental health and overall well-being as a main goal, (g) collaborative and competitive physical exercise activities and sports, (h) the participant’s awareness of the improvements through feedback and information, (i) mindful activities, (j) outdoor physical activities, especially in natural environments, (k) conducted and designed by multidisciplinary teams, (l) assessments using validated tests and scales, and (m) evaluation of long-term effects.

## Figures and Tables

**Table 1:** Author-informed recommendations for exercise programs included during digital detox.

	Recommendations for Digital Detox Programs	Physical Activity and Exercise Considerations to Be Included in Digital Detox Programs
Type of challenges	Detox challenges must be voluntary and self-selected according to the characteristics and motivations of participants	The selection of exercise type should consider participants’ characteristics and motivations, as various forms of exercise can be effective
Level of demand for the challenge	Reducing time is more recommended than total abstinence. Abrupt dopamine fasting (associated with total abstinence) may lead to negative consequences, so challenges must have different levels according to the baseline characteristics of the participants	Individualization is key and exercise intensity and volume must be adapted to the participant’s characteristics and physical fitness levels
Spatial and temporal restrictions	Changing settings like airplane mode or muting the notifications is useful. Restrictions may be related to a specific space (for instance, avoid using the smartphone in the bedroom) or time (for instance, avoid using the smartphone 1h before going to sleep)	Avoid using smartphones, wearables, and digital devices during exercise is recommended to pay full attention to the activity
Length of intervention	Effective length of intervention may range from a few days to several weeks	Exercise interventions may be more effective when the duration is ≥12 weeks, with at least 3 sessions per week
Promotion of healthy habits	It is important to integrate digital disconnection as a healthy habit in the daily living of the participant	Exercise interventions should aim to promote exercise adherence as part of an overall change towards a healthier lifestyle
Link between physical exercise and problematic use of the Internet and digital devices	Physical exercise should be considered as a core part of every digital detox program	Exercise interventions should target improvements in mental health and overall well-being to significantly contribute to digital detox programs
Social interactions	Digital detox programs must promote social interactions to avoid loneliness, as well as promote real interactions	Team sports and collaborative and competitive physical exercise activities are recommended.
Participants’ own awareness and goals	By using apps on Android or iPhone, participants can check their daily use of smartphones and establish adjusted individual goals (for instance, restricting the use of social media apps to 30 daily minutes)	Feedback and information must be provided to make the participant aware of the changes and improvements
Mindful activities	Activities aimed at increasing mindfulness are recommended	Mindful activities are recommended. The use of digital devices during exercise is not recommended to maintain full attention in the activity
Outdoor activities	Spending time outdoors might lead to further benefits compared to being indoor	Outdoor physical activities have shown larger benefits than indoor activities. Activities in natural environments, such as green and blue exercises, are recommended because of mental health and quality of life
Inter-disciplinary approach	The complexity of the topic may require cooperation of different professionals, including clinicians, psychologists, exercise professionals, etc.	Exercise professionals should design and conduct the exercise program. However, a multidisciplinary team is needed to assess improvements at all levels
Measuring tools	Utilization of validated scales and questionnaires is recommended. Those data may be complemented by data extracted directly from the user’s smartphone	Improvements must be assessed using validated tests and scales. A well-designed evaluation framework should be incorporated to measure the intervention’s effectiveness and to remodel future protocols
Long-term assessment	Studies must evaluate not only short-term but also long-term effects in order to check the retainment	The assessment of both chronic and acute effects of exercise is essential, but consistent follow-up is crucial to ensuring meaningful long-term changes

## Abbreviations

The following abbreviations are used in this manuscript:

DSM-5	Diagnostic and Statistical Manual of Mental Disorders
FoMo	Fear of Missing Out
